# Do we really need the “detriment” for radiation protection?

**DOI:** 10.1007/s00411-020-00861-y

**Published:** 2020-06-24

**Authors:** Joachim Breckow

**Affiliations:** Institute of Medical Physics and Radiation Protection (IMPS), University of Applied Sciences, THM, Giessen, Germany

**Keywords:** ICRP 103, Detriment, Radiation risk, Nominal risk coefficient, Incidence probability per dose, Mortality probability per dose, Damage function, Tissue weighting factor

## Abstract

The purpose of the ICRP detriment concept is to enable a quantitative comparison of stochastic radiation damage for the various organs. For this purpose, the organ-specific nominal risk coefficients are weighted with a function that is intended to express the amount of damage or, respectively, the severity of a disease. This function incorporates a variety of variables that do not depend on radiation parameters, but on characteristics of the disease itself. The question is raised as to whether the rather subtle way of defining the amount of damage is necessary for radiation protection purposes and whether a much simpler relationship can serve for this purpose as well or even better.

## The concept of detriment

Over a period of several decades and numerous steps of improvements, the ICRP succeeded in developing an elegant and sophisticated system of radiation protection which incorporates a multitude of parameters and values meant for an adequate description of radiation risk. One part of this ambitious and challenging system is the concept of “detriment” which is, however, not entirely without any conflicts (see, e.g., Müller et al. 2016). In the following, only one aspect of the entire problem will be dealt with. Particularly, this paper does not critically discuss the numbers given by ICRP for the different parameters involved.

The concept of detriment as described in ICRP 103 (2007) neither reflects a pure mortality risk model nor a pure incidence risk model. Instead, ICRP defines a weighted probability of harm (detriment) for each type of cancer, which takes into account both the likelihood of cancer increased by radiation exposure and certain “non-radiation” parameters such as the lethality of a cancer type, the loss of life expectancy, and the reduction of quality of life. The aim is to establish comparability between different types of cancers in terms of their severity and, thus, their “radiation relevance”. For example, despite probability of occurrence, thyroid cancer with a good prognosis is rated and weighted less than lung cancer with poor prognosis and high lethality. The damage-weighted risk per dose is referred to as “detriment” (unit: Sv^−1^).

The detriment defined by the ICRP in this way is the product of the probability of occurrence for a stochastic effect per dose, i.e., the nominal risk coefficient, and the degree of the effect’s severity (ICRP 2007; Cléro et al. 2019). While the risk coefficient depends on a variety of radiation-related parameters, the degree of severity does not.

## The measure of damage

The parameters that are involved in the determination of the risk coefficient, such as the model of the linear no-threshold dose–response relationship (LNT) or the dose and dose-rate effectiveness factor (DDREF), are subject of lasting debates in the radiation protection community and are in some aspects controversial. The ICRP definition of the detriment, particularly the measure of damage, on the other hand, is surprisingly little discussed and hardly questioned. This is even more remarkable because both, the way of including the radiation damage and the choice of values for the “damage parameters” (lethality, loss of life expectancy, and reduction of quality of life) can be done very differently. Thus, the way chosen by ICRP 103 (ICRP 2007) indicates some subjective components and means a certain assessment regarding the relevance or significance of radiation damage (Cléro et al. 2019). The ICRP detriment model represents one possible, but not the only possible measure of a damage-weighted risk. With respect to the choice of the various detriment parameters, the German Commission on Radiological Protection (SSK) recommends that “in the case of adjusting the DDREF … in parallel all of the other parameters pertaining to the detriment (should) be adapted to the latest scientific findings.” (SSK 2014).

The detriment *D*, i.e., the damage-weighted risk per dose, is defined according to ICRP 103 (ICRP 2007) as follows:

1 $$D = R_{I} \cdot (k + q \cdot (1 - k)) \cdot L = R_{I} \cdot d(k),$$

where:

$R_{I}$: Nominal risk coefficient, incidence probability per dose.

*k*: Lethality factor.

$R_{I} \cdot k$: Probability per dose of fatal cancer.

$R_{I} \cdot (1 - k)$: Probability per dose of non-fatal cancer.

*q*: Weighting function for the reduction of quality of life.

*L*: Relative loss of life expectancy.

*d*(*k*): Damage function.

A similar model based on mortality data had already been developed by ICRP 60 in its 1990 recommendations (ICRP 1991) to describe a weighted probability of damage. In ICRP 103 (ICRP 2007), this model has been refined and is now essentially based on incidence data, expressed by the nominal risk coefficient *R*_*I*_ with respect to a cancer type or, respectively, to the probability per dose for hereditary damage. The sum of all organ-specific detriments is the “total” detriment, which is given in ICRP (2007) for the whole population as 5.7% per Sv. This includes the detriment for hereditary damage with 0.1% per Sv, which, therefore, plays a minor role compared to that for cancers.

The weighting function *q*(*k*) according to ICRP (2007) includes the non-fatal damage, taking into account the reduction of the quality of life as a function of lethality *k*:

2 $$q(k) = q_{\min } + k \cdot (1 - q_{\min } ),$$

with *q*_min_ = 0.1 for all cancers except skin (*q*_min_ = 0) and thyroid (*q*_min_ = 0.2).

This relationship is intended to express the fact that even for a completely non-fatal cancer (*k* = 0), the quality of life can be reduced to a certain fraction given by *q*_min_ (*≠ *0).

All in all, this leads to a damage *d*(*k*) as a function of lethality *k* by the following expression:

3 $$d(k) = [(1 - q_{\min } ) \cdot (2k - k^{2} ) + q_{\min } ] \cdot L.$$

This expression, which seems not straightforward at first glance, has a considerable impact on the weighting of damage and, in turn, on the weighted risk coefficient as one of the main risk quantities in radiation protection. It may be due to the somewhat poorly traceable structure and the difficulty to interpret its meaning that the purpose or even the necessity of this function is so little discussed.

## Impact of the damage function *d*(*k*)

Essentially, the damage function Eq. (3) includes the parameters lethality factor *k* and relative loss of life expectancy *L*. For a given cancer site, *k* is defined as the quotient of the age-standardized mortality rate and the corresponding age-standardized incidence rate. The relative loss of life expectancy *L* is the life lost due to this cancer normalized by the average life lost over all cancers (for further detail see Breckow et al. 2018). Although *L* is not independent of *k,* it is, however, not expressed analytically as a function of *k* (at least not given explicitly in the ICRP formalism). *L* is the relative loss of life expectancy, i.e., relative to other organs. Thus, a low lethality factor (e.g., skin cancer: 0.002) must not mean necessarily a low relative life loss (skin cancer: 1.0) and vice versa (ICRP 2007).

**Fig. 1:**
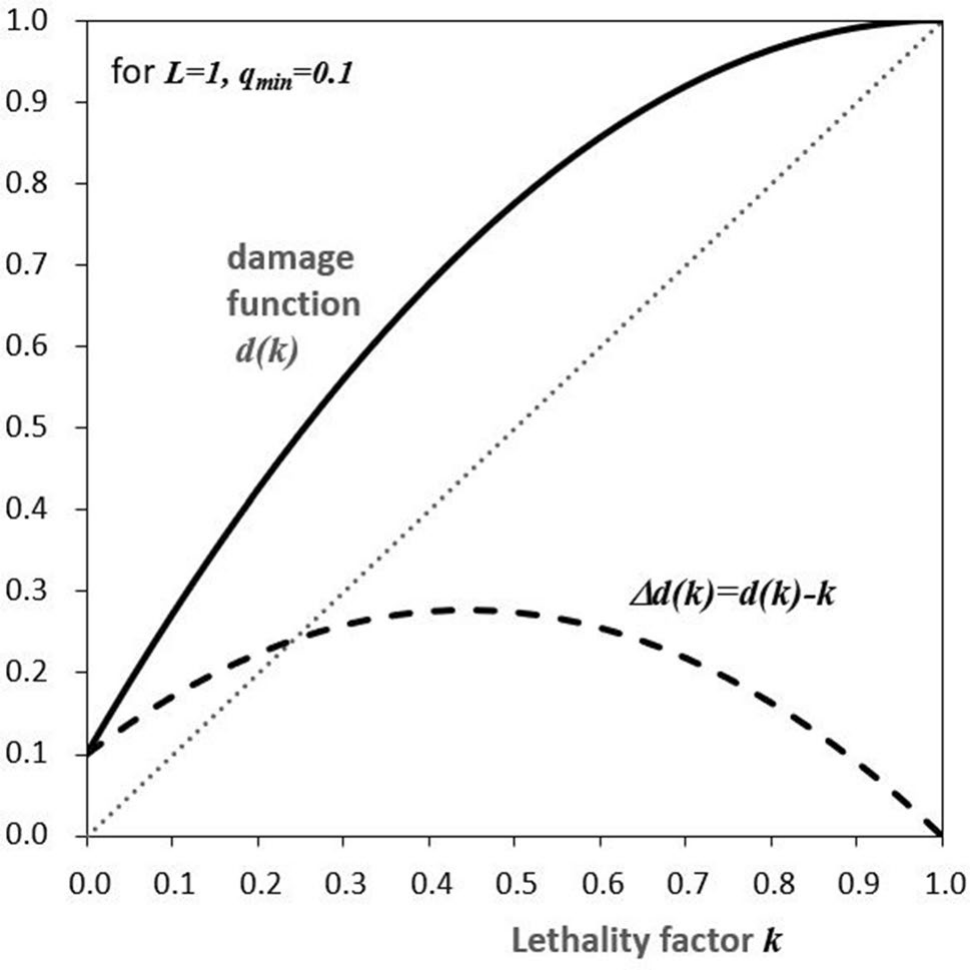
Damage *d*(*k*) (solid line) and non-fatal fraction *Δd*(*k*) (dashed line) as a function of the lethality factor *k* according to Eq. (3). The “minimum quality of life” is *q*_min_ = 0.1 and the relative loss of life expectancy is *L* = 1. The unweighted mortality fraction corresponds to the angle bisector *k* (dotted line). Modified from Breckow et al. (2018)

To discuss how the damage function of Eq. (3) affects the damage-weighted radiation risk, the course of *d*(*k*) is shown in Fig. 1 as a function of the lethality factor *k* and with (constant) relative loss of life expectancy *L* = 1. For clarity, in Eqs. (4)–(6) *L* is set equal to unity.

For a completely non-fatal cancer (*k* = 0), the damage corresponds to the “minimum quality of life” *q*_min_ and the detriment for this case is the *q*_min_-weighted incidence probability per dose:

4 $$D = R_{I} \cdot q_{\min } .$$

On the other hand, for a completely fatal cancer (*k* = 1), there is a weighting of *q* = 1 and the detriment results in:

5 $$D = R_{I} = k \cdot R_{I} = R_{M} .$$

Therefore, in the case of *k* = 1, the incidence probability per dose *R*_*I*_ is equal to the mortality probability per dose *R*_*M*_. For cancers with a lethality factor between 0 and 1, the detriment or, respectively, the damage function is composed of a lethal proportion *k* (angle bisector in Fig. 1) and a weighted non-lethal proportion (Fig. 1):

6 $$\Delta d(k) = d(k) - k.$$

With *L* = 1 the maximum contribution to the “non-fatal damage” is at *k* = 0.45 (45% mortality) and adds up to about 27% (Fig. 1). This means that the remarkably complex procedure of damage-weighting according to Eq. (3) provides only a small contribution to the total detriment.

A decisive parameter in the damage function *d*(*k*) according to Eq. (3) is the “minimum quality of life” *q*_min_. The larger *q*_min,_ the more restrictive a (non-fatal) cancer is considered and the stronger the associated reduction of quality of life is assessed. A larger *q*_min_ means a larger damage and, thus, a greater detriment with the same incidence probability. In addition, a larger *q*_min_ means a less pronounced dependence on *k*. Surprisingly, the ICRP makes little use of the possibility to reproduce this variability which, in principle, is incorporated in the detriment model: almost all organs are assigned the same value for *q*_min_ (see above).

To determine the impact of the individual cancers or organs on the evaluation of the damage function, one may consider Table 4.1a of Annex A from ICRP 103 (ICRP 2007). The corresponding values in Table 1 are taken for the total population. In the first column, *R*_*I*_ is the unweighted incidence probability per dose (nominal risk coefficients) for each cancer. Column 2 gives the corresponding lethality factor *k* and column 3 the damage-weighted incidence probability per dose *D* (detriment) calculated according to Eq. (1) (all values from ICRP 2007). From these values, the “non-lethal” proportion *Δd* by Eq. (6) was determined (column 4).

**Table 1:** Organ-specific parameters contributing to the detriment and to the damage function

Tissue	ICRP 103				*R*_*M*_* = R*_*I*_*·k*		
	(1) *R*_*I*_ [per 10,000 pers. per Sv]	(2) *k*	(3) *D* [per 10,000 pers. per Sv]	(4) *Δd*	(5) *R*_*M*_ [per 10,000 pers. per Sv]	(6) *R*_*M*_/*D*	(7) (*R*_*M*_/*D*)_*rel*_
Esophagus	15	0.93	13.0	− 0.06	14.0	1.07	1.48
Stomach	79	0.83	67.7	0.03	65.6	0.97	1.34
Colon	65	0.48	47.6	0.25	31.2	0.65	0.90
Liver	30	0.95	26.3	− 0.07	28.5	1.08	1.50
Lung	114	0.89	90.2	− 0.10	101.5	1.12	**1.56**
Bone	7	0.45	5.1	0.28	3.2	0.62	0.89
Skin	1000	0.002	4.0	0.00	2.0	0.50	0.71
Breast	112	0.29	78.8	0.41	32.5	0.41	0.57
Ovary	11	0.57	10.3	0.36	6.3	0.61	0.83
Bladder	43	0.29	16.7	0.10	12.5	0.75	1.03
Thyroid	33	0.07	13.3	0.33	2.3	0.17	**0.26**
Bone marrow	42	0.67	61.7	0.80	28.1	0.46	0.63
Gonads	20	0.80	25.4	0.47	16.0	0.63	0.89
Other solid	144	0.49	113.5	0.30	70.6	0.62	0.86
	**1715**		**573.6**		**414.3**	**0.69**	**1**

The highest (lung) and the lowest (thyriod) value in column (7) are highlighted (bold)

*R*_*I*_ incidence risk coefficient, *k* lethality factor, *D* detriment, *Δd* non-fatal fraction according Eq. (6), *R*_*M*_ Mortality risk coefficient. Columns (1)–(3) from ICRP (2007)

The minimum quality of life *q*_min_ and the relative loss of life expectancy *L* have only a small impact on the amount of damage, whereas the lethality factor has a very strong effect. This is in good agreement with sensitivity analyses by Zhang et al. (2020), who investigated the influence of various parameters on the detriment calculation.

Considering the minor impact of most of the damage parameters gives rise to the question of whether the rather subtle ICRP model representing the radiation damage according to Eq. (3) could be simplified to some degree. It could be proposed to replace Eq. (3) with its parameters lethality, reduction of quality of life, minimum quality of life and relative loss of life expectancy by the obviously rather simple proportionality to lethality *k*:

7 $$d(k) = [(1 - q_{\min } ) \cdot (2k - k^{2} ) + q_{\min } ] \cdot L\quad {\text{replaced by :}}\quad d(k) = k.$$

The detriment would then become nothing else than the simple unweighted mortality probability per dose, i.e., the mortality risk coefficient *R*_*M*_ (Table 1, column 5):

8 $$\begin{gathered} D = R_{I} \cdot (k + q \cdot (1 - k)) \cdot L\quad {\text{replaced by:}}\quad D = R_{I} \cdot d(k) = R_{I} \cdot k = R_{M} \hfill \\ \, \Rightarrow \;D = R_{M} . \hfill \\ \end{gathered}$$

The deviation of such a modified (in fact abolished) detriment from the current definition can be seen by relating both quantities for each organ, normalized to the total detriment (Table 1, column 7):

9 $$\left( {\frac{{R_{M} }}{D}} \right)_{rel} = \frac{{R_{M,rel} }}{{D_{rel} }} = {{\frac{{R_{M} }}{{R_{M,tot} }}} \mathord{\left/ {\vphantom {{\frac{{R_{M} }}{{R_{M,tot} }}} {\frac{D}{{D_{tot} }}}}} \right. \kern-\nulldelimiterspace} {\frac{D}{{D_{tot} }}}} = \frac{{R_{M} }}{D} \cdot \frac{573.6}{{414.3}}.$$

As can be seen from Table 1, columns 6 and 7, the differences between the mortality-based and the detriment model is for most organs in the order of few 10%. For lung cancer the deviation, is + 56% (Table 1, column 7) due to the comparatively low relative loss of life expectancy and for thyroid cancer, it is − 74% due to the exception of *q*_min_ = 0.2. With a view to the uncertainties of many of the radiation parameters and to the requirements of radiation protection, this variability seems to be tolerable.

Based on the organ-specific detriments, ICRP (2007) defined the tissue weighting factors *w*_*T*_ for the different organs as an essential part of the concept of the effective dose. More roughly than the detriments, these are grouped in only four classes with *w*_*T*_ values 0.01, 0.04, 0.08, and 0.12. To consider the impact of choosing the simple mortality-based model rather than the detriment model, in Table 2 the organ-specific relative detriment *D*_*rel*_ [cf. Eq. (9)] and the organ-specific relative mortality risk coefficient *R*_*M,rel*_ (relative to the corresponding tissue weighting factor *w*_*T*_) are compared. Although the deviations of the mortality-based model for some organs are somewhat larger, the general figure for most of the organs is quite the same as for the present detriment model. If the deviations, however, are judged (say, by ICRP or anyone else in the debate) to be too large, it would be rather straightforward with Table 1, column 5 to find a suitable set (say, as yet, grouped in four *w*_*T*_ classes) of tissue weighting factors, in the case one wants to choose a mortality-based weighting model instead of the detriment-based model. The most prominent deviations are for thyroid cancer and for lung cancer (see above). The mortality risk for lung cancer contributes to almost 25% to the total radiation-induced mortality risk (vs. about 16% based on detriment). However, if the larger contribution would be represented by a higher lung weighting factor, some far-reaching implications could occur. For example, the revision of the radon dose coefficient (ICRP 2014, 2018) means a modified conjunction between lung exposure by radon, the dose to the lung, and the risk for lung cancer. The detriment or, respectively, the weighting factor for lung is part of this conjunction. Since these parameters are identical for any kind of ionizing radiation, an alteration of the lung detriment or the lung weighting factor would equally change both, the lung risk coefficient for low-LET radiation and the dose coefficient for radon (Müller et al. 2016).

**Table 2:** Tissue weighting factors *w*_*T*_ (column 1) in comparison to the organ-specific relative detriment *D*_*rel*_ (column 2) from ICRP (2007) and to the organ-specific relative mortality risk coefficient *R*_*M,rel*_ (column 3) according to Eq. (8, replaced) and Eq. (9)

Tissue	(1) *w*_*T*_	(2) *D*_*rel*_	(3) *R*_*M,rel*_
Oesophagus	0.04	0.023	0.034
Stomach	0.12	0.118	0.158
Colon	0.12	0.083	0.075
Liver	0.04	0.046	0.069
Lung	0.12	0.157	0.245
Bone	0.01	0.009	0.008
Skin	0.01	0.007	0.005
Breast	0.12	0.139	0.078
Bladder	0.04	0.029	0.030
Thyroid	0.04	0.022	0.006
Bone marrow	0.12	0.107	0.068
Gonads	0.08	0.044	0.039

## Conclusion

The ICRP detriment is a useful tool for defining a measure of damage which considers, in addition to lethality, non-fatal contributions as well. It offers the possibility to compare different cancers or organs in terms of their contribution to the total risk. The degree of damage represented in the detriment model by a weighting function (Eq. 1) does not depend on any radiation parameters. It is subject to a temporal trend that reflects the improved prognosis through advances in cancer diagnosis and therapy (Breckow et al. 2018). This means that the detriment, i.e., the “radiation risk” may decrease over time, even if the nominal risk coefficient remains unchanged. This circumstance is not without problems for acceptance and may raise questions as to whether this type of definition of detriment adequately represents radiation risk. The accusation is quite conceivable that one wants to instrumentalize medical progress in such a way that the radiation risks are “calculated downward”.

The damage function includes several parameters that are intended to represent the severity of a (cancer) disease. However, other values, other parameters, or even other models could be conceivable, such as the severity of cancer therapy and its side effects, which could be incorporated into a model as well. However, not only a “refinement” of the model, but on the contrary also a coarser model could fulfill the intended purpose of a detriment concept. A suitable model that aims in this direction could be the model described above, which only considers the mortality as the one and only parameter included. A less sophisticated model like this would possibly allow for a clearer and more straightforward description of risk assessment for radiation protection purposes. With respect to a traceable and transparent concept of radiation protection which should be intended anyway, it could be considered as favorable.

The ICRP detriment model serves, among others, the purpose of laying the foundation for the concept of the effective dose by determining the tissue weighting factors *w*_*T*_. The *w*_*T*_ values for the different organs roughly represent the ratio of the organ-specific detriment to the total detriment. However, these ratios, as well as other characteristics of the effective dose, would not considerably change by choosing the “mortality model” instead of the detriment model. The quantitative comparison of the (stochastic) damage in the different organs would also remain almost unchanged.

The concepts of both, the effective dose and the detriment, are for radiation protection purposes only and are not suitable to carry out risk estimates for individuals or special populations (e.g., ICRP 2007). It is true that dose limits are based on risk assessments. However, the risks that are considered as to be “tolerable” and are consequently linked to dose limits are not determined, neither in radiation protection nor in other areas of environmental and occupational protection, on the basis of a detriment, but as a rule by considering mortality data (e.g., Rühm et al. 2020). Various damage-weighted risk variables are known in environmental and occupational protection, such as the concept of DALY (Disability Adjusted Life Years) of the World Bank and the WHO (e.g., Chen et al. 2015). The ICRP concept of detriment, however, is applied nowhere outside of radiation protection. Thus, at least for this reason, with this concept, no comparability with carcinogenic substances from other areas can be achieved.

Against this background, for almost all purposes of radiation protection, there would be no need to use a subtle model according to Eq. (3) to consider radiation damage in any way. The concept of detriment certainly is of some elegance and seems to reflect adequately the contributions of the different organs to radiation-induced risk. However, this subtlety comes at the expense of the transparency and comprehensibility of risk assessments in radiation protection. The question is whether we really need a fine-tuning like this for the purposes of radiation protection.

Our radiation protection system works quite well. It would, however, probably work as well and without any restriction even without (or with a simpler) detriment model. On the other hand, continuity in radiation protection is an important criterion. Thus, even if one were to think that radiation protection could be done without detriment, this would not necessarily mean that one had to make a plea for it to be abolished.
